# Socioeconomic disparities in HPV vaccine uptake: multivariable analysis of vaccination data from Tianjin (2018–2023)

**DOI:** 10.3389/fpubh.2025.1428267

**Published:** 2025-02-27

**Authors:** Jing Xiang, Xuan Sun

**Affiliations:** ^1^Gynecology Department, Nankai University Hospital, Tianjin, China; ^2^Zhou Enlai School of Government, Nankai University, Tianjin, China

**Keywords:** HPV vaccines, vaccine equity, socio-economic status, multivariable logistic regression, urban–rural disparities

## Abstract

**Objectives:**

As the first socio-demographic profiling of HPV vaccines in Chinese cities, this study assesses equity implications through compositional analysis of covered populations, with multilevel examination of vaccine-type selection determinants.

**Method:**

Utilizing HPV vaccination data obtained from the Jinnan Center for Disease Control and Prevention (CDC) spanning from 2018 to 2023, we conducted a retrospective analysis. Hierarchical logistic regression was employed to model the joint effects of age, ethnicity, occupation, and urban–rural residence on vaccination behaviors. Vaccine type preference was categorized as bivalent, quadrivalent, or nonavalent.

**Result:**

Three key disparities were revealed in the analysis. Age-stratified access revealed the highest proportion of recipients among women aged 33–38 years (29.6%) and 39–44 years (21.9%), contrasting with less than 1% participation in the 9–14 year-old cohort. Educationally, 87.3% held at least a bachelor’s degree, compared to 12.7% with below-college education (*χ*^2^ = 6048.89, *p* < 0.001). Clear urban–rural divide, with 99.7% of recipients in urban areas and just 0.3% in rural areas (*χ*^2^ = 76.79, *p* < 0.001). Vaccine-type selection showed socioeconomic patterns, with nonavalent vaccines preferred by urban professionals (OR = 1.577, 95% CI: 1.16–2.142) and those with incomes above 5000 yuan (OR = 1.958, 95% CI: 0.26–3.527).

**Conclusion:**

Demonstrating Hart’s Inverse Care Law, Tianjin’s program disproportionately immunizes socioeconomically secure urbanites. We propose: (1) school-based mandates for pre-sexual debut cohorts; (2) rural vaccination-social insurance integration; (3) domestic 9-valent vaccine development with needs-based subsidies. These evidence-based reforms are critical for achieving equitable 90% coverage by 2030.

## 1 Introduction

Cervical cancer, primarily caused by persistent infection with high-risk types of HPV, remains a significant global health issue. In 2020, the disease resulted in over 600,000 new cases and 341,831 deaths worldwide (1), disproportionately affecting socioeconomically disadvantaged populations, particularly in low- and middle-income countries. These disparities arise from barriers such as limited healthcare access, low health literacy, and financial constraints (2–4). Despite substantial advances in cervical cancer prevention through HPV vaccination, socio-economic and geographical inequalities continue to hinder vaccine accessibility and uptake, particularly in developing countries like China.

In China, the incidence of cervical cancer has increased by 3.5% from 2018 to 2020, with 109,700 new cases, accounting for 5.2% of all female cancer cases (5). HPV types 16 and 18 are responsible for approximately 70% of cervical cancer cases (6), with other high-risk types such as 31, 33, 45, 52, and 58 contributing significantly (7, 8). HPV vaccination programs have proven to be effective in reducing the prevalence of HPV infections and the incidence of cervical cancer (9, 10). The introduction of bivalent, quadrivalent (4-Valent), and nonavalent (9-Valent) vaccines has been particularly successful in preventing HPV-related cancers globally (11, 12).

However, despite these advances, China faces stark disparities in HPV vaccination coverage, with only 2.24% of the female population vaccinated by 2020 (13). This low vaccination rate highlights significant gaps in public health efforts, particularly in terms of accessibility and education (14). Socio-economic factors such as age, education, and occupation contribute to inequities in vaccine uptake, leading to a paradox where socioeconomically secure urban populations are more likely to be vaccinated, while higher-risk groups, including younger and rural populations, remain under-vaccinated.

Tianjin, a rapidly developing municipality in northern China, provides an ideal case study for exploring these challenges. Jinnan District, one of Tianjin’s key districts, is particularly notable for its demographic diversity and socio-economic stratification. According to the 2020 census, Jinnan District’s population stands at 928,066, with women comprising 47.14% and the working-age group (15–59 years) making up 70.61% (15). A substantial portion of the female population is within the age range eligible for HPV vaccination (9–45 years), making Jinnan a relevant site for studying HPV vaccination patterns.

This study seeks to assess the socio-economic attributes and geographical distribution of the HPV vaccination population in Jinnan District. The objective is to analyze the composition of the currently vaccinated cohort and identify the key factors influencing vaccine type preferences, ultimately providing valuable insights for the future enhancement of vaccination promotion strategies and reducing cervical cancer incidence in the region.

## 2 Materials and methods

### 2.1 Ethics approval, study population

This study was approved by the Nankai University Biomedical Ethics Committee (Approval No. NKU-IRB-2023141). Informed consent was not required for the retrospective analysis, as all data were anonymized and fully de-identified to ensure privacy, with access restricted to authorized personnel. The dataset, which includes both vaccination records and socio-demographic information (age, occupation, marital status, and geographic location), was provided by the Tianjin Jinnan District CDC as part of the city’s routine public health surveillance system. The data covered the period from January 2018 to June 2023 and pertained to women residing in the Jinnan District of Tianjin who participated in the vaccination program. A formal request was submitted to the CDC for the data, which was commissioned specifically for this study.

### 2.2 Data collection and study procedure

To ensure the validity and reliability of the study, the data collection process adhered to a systematic procedure:

- Data Collection: The Tianjin Jinnan District CDC collected and maintained vaccination records and socio-demographic information through its routine public health surveillance system, which includes both electronic health records and paper-based records.
- Data Anonymization: The CDC anonymized the data to ensure participant privacy and confidentiality.
- Data Transfer: The anonymized dataset was transferred to the research team for analysis.
- Data Cleaning and Preparation: The research team conducted data cleaning and preparation to ensure the dataset was ready for analysis.

### 2.3 Statistical analysis

IBM SPSS Statistics 25.0 was used for data analysis. Categorical variables were summarized as frequencies and percentages, and continuous variables as means and standard deviations. Associations between socio-demographic factors and vaccine type preference were assessed using chi-square tests. Collinearity was assessed using variance inflation factors (VIFs), and final variables for the model were selected accordingly. Logistic regression identified predictors of vaccine type preference, with statistical significance set at *p* < 0.05.

Age was initially grouped into seven five-year brackets for descriptive statistics but was reclassified into four broader categories (9–19, 20–29, 30–39, 40–50 years) in the regression analysis due to small sample sizes in the youngest and oldest groups. Results are presented in tables and charts with narrative explanations.

## 3 Results

### 3.1 Socio-demographic characteristics

The study cohort comprised 33,002 female HPV vaccine recipients, with a mean age of 33.0 years (SD = 7.6). Detailed socio-demographic characteristics are presented in Table 1. The majority of recipients were urban residents (99.7%), with 85.1% married and 99.5% of Han ethnicity. Educational attainment was high, with 87.3% holding at least a bachelor’s degree. Occupation-based categorization showed that homemakers (25.8%) were the largest group, followed by labor-intensive workers (13.7%) and students (13.3%). Income levels varied, with 46.7% earning between 5,000 and 7,000 RMB per month.

**Table 1:** Socio-demographic characteristics, distribution of vaccine type.

Variable	N (100%)	Cervarix	Cecolin	Gardasil 9	Gardasil 4	χ^2^	p
Age group (years)						20174.793	<0.001
9–14	238 (0.7%)	23 (9.4%)	145 (59.4%)	76 (31.1%)	–		
15–20	1,659 (5.0%)	49 (2.1%)	190 (8.1%)	2,084 (89.5%)	3 (0.1%)		
21–26	6,030 (18.3%)	14 (0.2%)	657 (12.2%)	4,664 (87.0%)	22 (0.4%)		
27–32	6,442 (19.5%)	205 (3.1%)	2,729 (42.3%)	1,773 (27.5%)	1,735 (26.9%)		
33–38	9,776 (29.6%)	440 (4.5%)	5,458 (55.8%)	377 (3.8%)	3,501 (35.8%)		
39–44	7,215 (21.9%)	517 (7.1%)	4,397 (60.9%)	186 (2.5%)	2,115 (29.3%)		
45–50	1,642 (5.0%)	223 (13.5%)	1,071 (65.2%)	10 (0.6%)	338 (20.5%)		
Education degree▱						6048.89	<0.001
Compulsory education	1,457 (4.4%)	52 (3.5%)	291 (19.9%)	1114 (76.4%)	–		
Below bachelor’s degree	436 (1.3%)	85 (19.4%)	92 (21.1%)	130 (29.8%)	129 (29.5%)		
Higher education	1,567 (4.7%)	19 (1.2%)	125 (7.9%)	1374 (87.6%)	49 (3.1%)		
Bachelor	28,797 (87.3%)	1,214 (4.2%)	14,114 (49.0%)	6,304 (21.8%)	7,165 (24.8%)		
Post-bachelor degree	745 (2.3%)	101 (13.5%)	25 (3.3%)	248 (33.2%)	371 (49.7%)		
Marital status						7586.46	<0.001
Married	28,075 (85.1%)	1,348 (4.8%)	13,912 (49.6%)	5,283 (18.8%)	7,532 (26.8%)		
Single	4,927 (14.9%)	123 (2.4%)	735 (14.9%)	3,887 (78.8%)	182 (3.6%)		
Ethnic group						103.544	<0.001
Non-Han	151 (0.5%)	27 (17.8%)	22 (14.5%)	64 (42.3%)	38 (25.1%)		
Han	32,851 (99.5%)	1,444 (4.3%)	14,625 (44.5%)	9,106 (27.7%)	7,676 (23.3%)		
Residence						76.794	<0.001
Rural	104 (0.3%)	18 (17.3%)	12 (11.5%)	48 (46.1%)	26 (25.0%)		
Urban	32,898 (99.7%)	1,453 (4.4%)	14,635 (44.5%)	9,122 (27.7%)	7,688 (23.3%)		
Occupation						8027.227	<0.001
Freelancers (FLC)	2,137 (6.5%)	60 (2.8%)	1,192 (55.8%)	600 (28.1%)	285 (13.3%)		
Homemakers (HM)	8,527 (25.8%)	458 (53.7%)	4,011 (47.0%)	1,807 (21.2%)	2,251 (26.4%)		
Intensive labor personnel (ILP)	4,529 (13.7%)	182 (4.0%)	2,643 (58.4%)	565 (12.5%)	1,139 (25.1%)		
Personnel ensuring basic social services (PEBSS)	3,658 (11.1%)	149 (40.7%)	1,980 (54.1%)	577 (15.8%)	952 (26.0%)		
Service personnel (SP)	4,230 (12.8%)	168 (39.7%)	2,226 (52.6%)	774 (18.3%)	1,062 (25.1%)		
State-owned enterprise personnel (SOEP)	1,809 (5.5%)	113 (62.4%)	620 (34.3%)	333 (3.6%)	743 (9.6%)		
Students (STD)	4,374 (13.3%)	90 (6.1%)	691 (4.8%)	3,498 (38.2%)	95 (1.3%)		
Technical personnel (TP)	3,738 (11.3%)	251 (6.7%)	1,284 (34.3%)	1,016 (27.1%)	1,187 (31.7%)		
Monthly income (RMB)						7534.961	<0.001
1500–2500	4,552 (13.8%)	93 (4.7%)	823 (18.0%)	3496 (76.8%)	140 (3.0%)		
2500–5000	10,859 (32.9%)	320 (2.9%)	6,322 (58.2%)	1,996 (18.3%)	2,221 (20.4%)		
5000–7000	15,422 (46.7%)	974 (6.3%)	6,885 (44.6%)	3,067 (19.8%)	4,496 (29.1%)		
7000–10000	1,950 (5.9%)	81 (4.1%)	531 (27.2%)	595 (30.5%)	743 (38.1%)		
Sum	33,002	1,471 (4.5%)	14,647 (44.4%)	9,170 (27.8%)	7,714 (23.4%)		

▱ Education degree represents the highest level of formal education attained by the participants.

**Figure 1:**
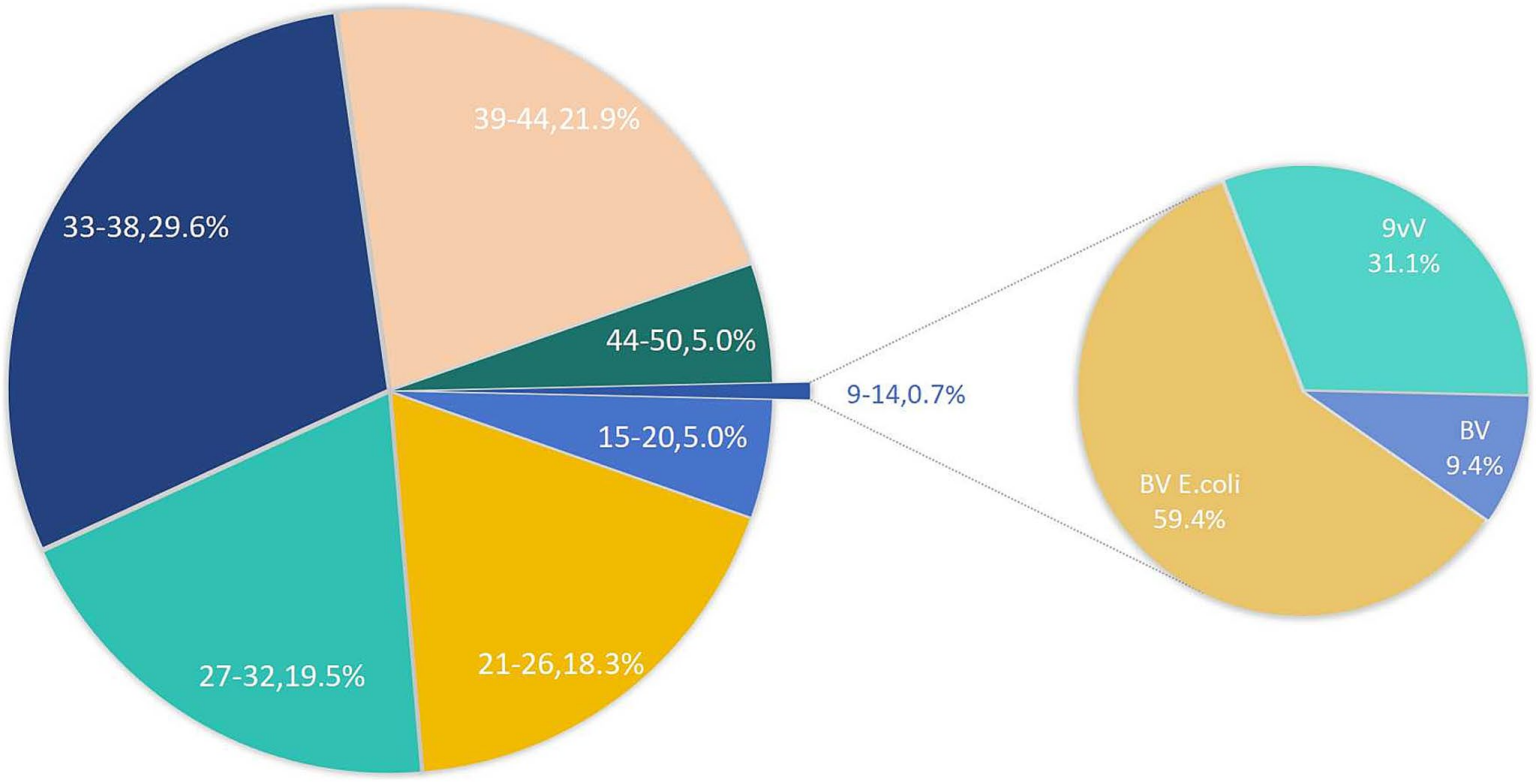
Age range of HPV vaccination and type distribution for ages 9–14.

Figure 1 presents the vaccine adoption trends among various age groups. Notably, the adoption rate for the 9–14 age group is strikingly low, standing at merely 0.7%. Interestingly, within the 45–50 age bracket, 5% of individuals have initiated vaccination, beyond the recommended age range. Among the youngest cohort, the *E. coli*-based bivalent vaccine emerges as the most preferred option, constituting 59.4% of vaccinations, with the 9-valent vaccine following closely at 31.1%.

### 3.2 HPV vaccine type distribution

The HPV vaccine’s annual response rate has significantly and cumulatively increased over time (Table 2). Initially dominated by the quadrivalent Gardasil 4 (2018–2020), which accounted for 50–80% of doses, a major shift occurred in 2021 when the domestic Cecolin bivalent vaccine captured 63.7% of doses by 2022, driven by policy incentives and cost-effectiveness. Despite growing availability, the 9-valent vaccine remained a minority choice, representing just 21–33% of doses.

**Table 2:** The annual distribution of HPV vaccine doses and completion rates.

Categories	Total dose	Cervarix	Cecolin	Gardasil9	Gardasil4
Year					
2018	280 (0.3%)	56 (20.0%)	–	–	224 (80.0%)
2019	596 (0.7%)	174 (29.1%)	103 (17.2%)	319 (53.5%)	–
2020	6,277 (7.2%)	1,239 (19.7%)	84 (1.3%)	1,810 (28.8%)	3,144 (50.0%)
2021	14,907 (17.0%)	1,251 (8.3%)	5,069 (34.0%)	4,658 (31.2%)	3,929 (26.3%)
2022	46,722 (53.4%)	975 (2.0%)	29,769 (63.7%)	10,168 (21.7%)	5,810 (12.4%)
2023	18,778 (21.5%)	499 (2.6%)	6,442 (34.3%)	6,153 (32.7%)	5,684 (30.2%)
Programme completion					
Second dose	29,489 (89.36%)	1,419 (4.8%)	14,036 (47.5%)	7,523 (25.5%)	6,511 (22.0%)
Third dose	25,064 (75.95%)	1,303 (5.1%)	12,681 (50.5%)	6,197 (24.7%)	4,883 (19.4%)
Fourth dose	5 (0.02%)	1 (20.0%)	–	2 (40.0%)	2 (40.0%)
Total dose	87,560	4,194 (4.7%)	41,364 (47.2%)	19,110 (21.8%)	22,892 (26.1%)

Completion rates showed critical attrition: 89.4% received the second dose, but only 75.95% completed the full three-dose regimen, with minimal uptake for the fourth dose (0.02%). Among the five extra-protocol vaccinations, four were delayed catch-up doses, and one involved switching vaccine types.

**Figure 2:**
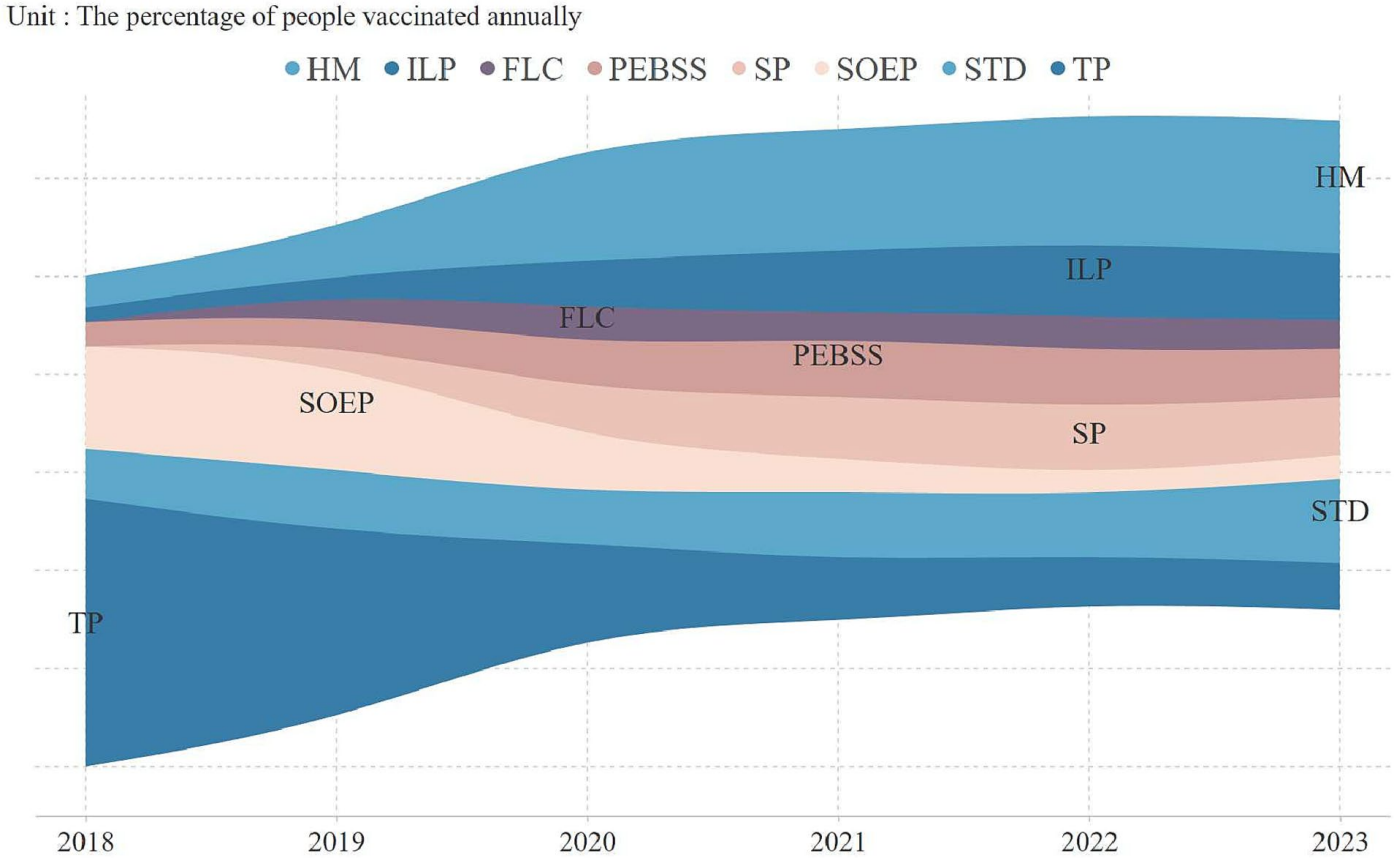
Yearly vaccination coverage by occupational categories.

**Figure 3:**
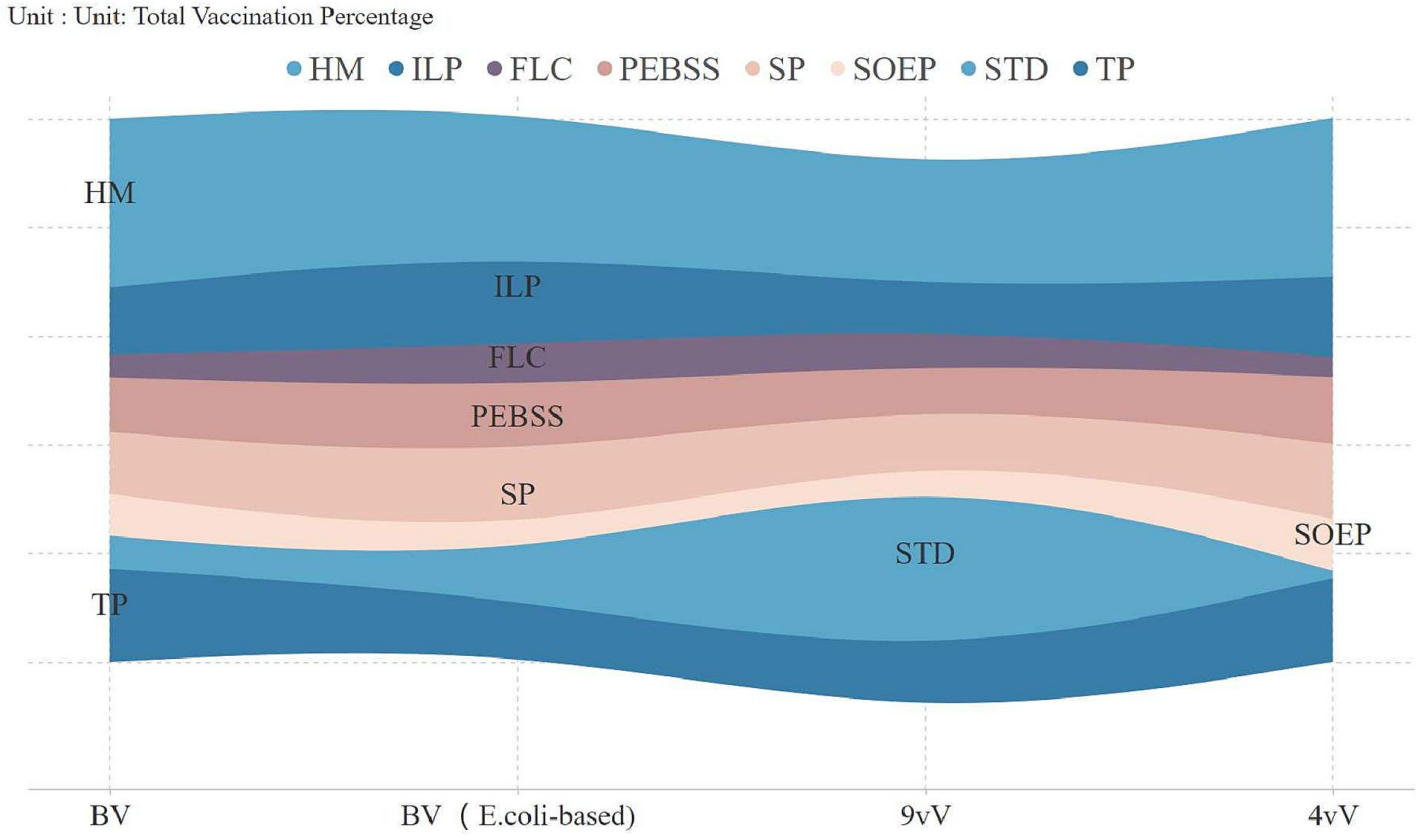
Distribution of vaccination types across occupational categories.

An analysis of vaccine uptake by occupation revealed key trends (Figure 2). In 2018, technical professionals led adoption (54.7%), but by 2020, homemakers became the largest group, comprising 25% of vaccinations, continuing into subsequent years. Vaccination rates among technical professionals and state-owned enterprise employees significantly declined, reaching 9.6 and 4.9% by 2023. Figure 3 highlights vaccine preferences across occupations. Homemakers showed broad vaccine type preferences, technical professionals displayed a similar pattern. In contrast, students preferred the 9-valent vaccine, with 38.2% selecting it, marking them as a key consumer group for this vaccine.

### 3.3 Key predictors of vaccine type preference

#### 3.3.1 The chi-square test

The analysis began with chi-square tests to examine the relationships between independent variables and vaccine type preference. Significant results (*p* < 0.001) were found for all variables, justifying their inclusion in the regression analysis. Key variables such as Age Group (*χ*^2^ = 20174.793), Education Level (*χ*^2^ = 6048.89), and Marital Status (*χ*^2^ = 7586.46) showed strong significance (Table 1). These results confirm their relevance as predictors of vaccine preference.

#### 3.3.2 Collinearity and model fit assessment

Before regression analysis, collinearity diagnostics were conducted (Table 3). Moderate collinearity between education and marital status led to their exclusion. The remaining variables showed satisfactory tolerance (all >0.8) and VIF < 1.5, confirming minimal multicollinearity and suitability for the model. Model adequacy was confirmed by Pearson’s chi-square and Nagelkerke’s pseudo R-squared, both indicating strong significance. The likelihood ratio test highlighted the key roles of Occupational Category and Monthly Income/Expenditure in predicting vaccine preference, while variables like Ethnicity and Place of Residence, though less significant, were retained for a more comprehensive analysis (Table 4).

**Table 3:** Collinearity statistics.

Variable	B	S.E.	Beta	t	p	95% CI	Tolerance	VIF
Constant	2.871	0.021		135.384	<0.001	[2.829, 2.913]		
Ethnic group	−0.048	0.071	−0.004	−0.68	0.497	[−0.188, 0.091]	0.966	1.035
Residence	0.04	0.086	0.003	0.468	0.64	[−0.128, 0.208]	0.967	1.034
Occupation	0.034	0.002	0.088	15.462	<0.001	[0.03, 0.039]	0.897	1.115
Monthly income	0.15	0.006	0.141	23.773	<0.001	[0.137, 0.162]	0.826	1.21
Age	−0.176	0.006	−0.165	−27.612	<0.001	[−0.188, −0.163]	0.82	1.22

Independent variable: Type Of Vaccine.

B shows the change in the dependent variable for a one-unit change in the independent variable. Beta compares the strength of predictors in standard deviations.

**Table 4:** Likelihood ratio test and model fit metrics.

Effect	−2 log likelihood	*χ* ^2^	df	p
Intercept	2052.467a	0	0	
Occupation	2695.901	643.434	21	<0.001
Monthly income	2899.862	847.395	12	<0.001
Age	16797.15	14744.683	9	<0.001
Residence	2085.34	32.873	3	<0.001
Ethnic group	2106.74	54.273	3	<0.001
Goodness-of-fit indicators	Pearson’s Chi-Squared	1292.8		<0.001
Nagelkerke R-squared				0.558

A *p*-value < 0.001 indicates a significant model fit. Degrees of freedom.

#### 3.3.3 The regression model

Logistic regression identified age as the strongest predictor of vaccine selection. Younger individuals (20–29 years) showed extraordinary preference for the 9-valent vaccine (OR = 405.99, 95% CI: 296.5–555.9, *p* < 0.001), whereas older recipients (30–39 years) favored domestic bivalent or quadrivalent options (OR = 1.62–2.50, *p* < 0.001).

Ethnicity and urbanization compounded these trends: Han Chinese (OR = 8.87, *p* < 0.001) and urban residents (OR = 7.29, *p* < 0.001) disproportionately chose domestic vaccines, reflecting both accessibility and cultural trust in locally produced biologics. Occupation-mediated disparities persisted, with public sector employees leaning toward 9-valent vaccines (OR = 1.58, *p* < 0.05) and freelancers/essential workers preferring domestic options (OR = 2.24–2.94, *p* < 0.001) (Table 5).

**Table 5:** Logistic regression for vaccine type selection.

Parameter	Estimate (B)	S.E.	df	p	OR (95% CI)
BV (E. coli-based)					
Intercept	−1.787	0.757	1	<0.05	–
Age group = 9–19	0.293	0.248	1	0.237	1.341 (0.825–2.179)
Age group = 20–29	1.217	0.141	1	<0.001	3.375 (2.562–4.447)
Age group = 30–39	0.482	0.059	1	<0.001	1.62 (1.443–1.818)
Age group = 40–50	Ref.	–	0	–	–
Ethnic Group = Han	2.182	0.307	1	<0.001	8.866 (4.858–16.18)
Ethnic Group = Non-Han	Ref.	–	0	–	–
Residence = Urban	1.986	0.395	1	<0.001	7.286 (3.359–15.806)
Residence = Rural	Ref.	–	0	–	–
Occupation = Freelancing	1.077	0.152	1	<0.001	2.935 (2.18–3.951)
Occupation = Homemaker	0.259	0.09	1	<0.05	1.295 (1.085–1.546)
Occupation = Intensive Labor Personnel	0.484	0.113	1	<0.001	1.623 (1.3–2.026)
Occupation = Personnel Ensuring Basic Social Service	0.805	0.111	1	<0.001	2.238 (1.801–2.781)
Occupation = Service Personnel	0.459	0.112	1	<0.001	1.582 (1.27–1.971)
Occupation = State-Owned Enterprise Personnel	0.107	0.124	1	0.39	1.113 (0.872–1.419)
Occupation = Student	−1.08	0.437	1	<0.05	0.34 (0.144–0.8)
Occupation = Technical Personnel	Ref.	–	0	–	–
Monthly Income = 1500–2500	1.189	0.716	1	<0.001	1.208 (0.297–4.913)
Monthly Income = 2500–5000	−0.203	0.595	1	0.733	0.816 (0.254–2.62)
Monthly Income = 5000–7000	−1.085	0.593	1	0.067	0.338 (0.106–1.08)
Monthly Income = 7000–10000	−1.226	0.603	1	<0.05	0.294 (0.09–0.957)
Monthly Income = 10000–15000	Ref.	–	0	–	–
9-Valent Vaccine					
Intercept	−4.647	0.784	1	<0.001	–
Age group = 9–19	4.578	0.254	1	<0.001	97.329 (59.218–159.969)
Age group = 20–29	6.006	0.16	1	<0.001	405.992 (296.533–555.857)
Age group = 30–39	1.383	0.106	1	<0.001	3.988 (3.24–4.909)
Age group = 40–50	Ref.	–	0	–	–
Ethnic Group = Han	1.907	0.299	1	<0.001	6.733 (3.747–12.097)
Ethnic Group = Non-Han	Ref.	–	0	–	–
Residence = Urban	1.146	0.367	1	<0.05	3.146 (1.532–6.46)
Residence = Rural	Ref.	–	0	–	–
Occupation = Freelancing	0.218	0.173	1	0.206	1.244 (0.887–1.744)
Occupation = Homemaker	−0.043	0.115	1	0.711	0.958 (0.764–1.201)
Occupation = Intensive Labor Personnel	−0.163	0.143	1	0.253	0.85 (0.643–1.123)
Occupation = Personnel Ensuring Basic Social Service	0.097	0.137	1	0.48	1.102 (0.842–1.442)
Occupation = Service Personnel	0.018	0.137	1	0.894	1.019 (0.778–1.333)
Occupation = State-Owned Enterprise Personnel	0.455	0.156	1	<0.05	1.577 (1.16–2.142)
Occupation = Student	−0.414	0.462	1	0.37	0.661 (0.267–1.635)
Occupation = Technical Personnel	Ref.	–	0	–	–
Monthly Income = 1500–2500	0.621	0.791	1	0.433	1.86 (0.395–8.772)
Monthly Income = 2500–5000	0.386	0.668	1	0.564	1.471 (0.397–5.447)
Monthly Income = 5000–7000	1.043	0.665	1	<0.001	1.958 (0.26–3.527)
Monthly Income = 7000–10000	1.617	0.677	1	<0.001	2.853 (0.491–6.985)
Monthly Income = 10000–15000	Ref.	–	0	–	–
4-Valent Vaccine					
Intercept	1.964	0.711	1	<0.05	–
Age group = 9–19	−22.994	0	1		1.03E-10
Age group = 20–29	0.792	0.148	1	<0.001	2.207 (1.651–2.95)
Age group = 30–39	0.915	0.061	1	<0.001	2.497 (2.215–2.816)
Age group = 40–50	Ref.	–	0	–	–
Ethnic Group = Han	1.02	0.275	1	<0.001	2.774 (1.618–4.754)
Ethnic Group = Non-Han	Ref.	–	0	–	–
Residence = Urban	0.413	0.337	1	0.22	1.512 (0.781–2.927)
Residence = Rural	Ref.	–	0	–	–
Occupation = Freelancing	−0.307	0.162	1	0.058	0.736 (0.536–1.011)
Occupation = Homemaker	−0.181	0.093	1	0.05	0.834 (0.696–1.0)
Occupation = Intensive Labor Personnel	0.079	0.116	1	0.497	1.082 (0.861–1.36)
Occupation = Personnel Ensuring Basic Social Service	0.201	0.114	1	0.078	1.222 (0.978–1.528)
Occupation = Service Personnel	0.097	0.115	1	0.399	1.102 (0.879–1.38)
Occupation = State-Owned Enterprise Personnel	0.337	0.124	1	<0.05	1.401 (1.099–1.785)
Occupation = Student	−1.43	0.459	1	<0.05	0.239 (0.097–0.589)
Occupation = Technical Personnel	Ref.	–	0	–	–
Monthly Income = 1500–2500	−1.781	0.722	1	<0.05	0.168 (0.041–0.694)
Monthly Income = 2500–5000	−2.06	0.592	1	<0.05	0.127 (0.04–0.407)
Monthly Income = 5000–7000	−2.436	0.589	1	<0.001	0.087 (0.028–0.278)
Monthly Income = 7000–10000	−1.644	0.599	1	<0.05	0.193 (0.06–0.625)
Monthly Income = 10000–15000	Ref.	–	0	–	–

OR (Odds Ratio) measures the association between exposure and outcome. The 95% CI shows the range within which the true Odds Ratio is likely to fall with 95% confidence.

## 4 Discussion

This comprehensive analysis of HPV vaccination patterns in China reveals critical socio-demographic and economic determinants shaping vaccine uptake and type preferences, offering novel insights into public health challenges in a rapidly evolving healthcare landscape. While previous studies have predominantly focused on regional prevalence and vaccination willingness, our findings highlight the complex interplay of age, urban–rural disparities, ethnicity, and socio-economic status in driving inequitable vaccine distribution—a phenomenon increasingly recognized in global contexts but underexplored in China’s unique socio-cultural environment.

Age composition in the vaccinated population emerged as a significant issue, with a severe imbalance observed across different age groups. Women aged 33 and older demonstrated the highest vaccination proportion, reflects greater health awareness, economic stability, and improved healthcare access in this age group. In contrast, vaccination participation were notably low among the 9–14 age group—the optimal target for HPV vaccination (16, 17). More than half of these adolescents preferred the domestic bivalent vaccine, driven by financial constraints rather than the most protective options (18, 19). This preference highlights the financial barriers faced by families, especially in rural and economically disadvantaged areas, where transportation costs and out-of-pocket expenses limit timely access to vaccination (20). Limited healthcare infrastructure and a lack of health education about the long-term benefits of HPV vaccination further delay decision-making, particularly among parents who prioritize immediate financial concerns over preventive health (21). Furthermore, the participation rate among women aged 15–26, who are eligible for catch-up vaccinations, was only 23.3%. This confirms a significant delay in vaccine adoption, consistent with previous studies, particularly among younger populations (22, 23), Economic pressures and a lower sense of urgency regarding preventive healthcare appear to be the primary contributors to this delay.

Epidemiological studies in Jilin, China have shown the widespread nature and genetic diversity of HPV, including high-incidence strains such as HPV16, HPV52, and HPV58. These studies highlight a bimodal age-specific prevalence, with peaks in the 30–34 and 55–59 age groups (24). Early vaccination, particularly before sexual debut (i.e., the 9–14 age group), remains the most effective preventive strategy to reduce the incidence of HPV-related diseases in these high-risk age groups. Studies also manifest that the antibody response in the 9–14 age group is twice as high as in individuals aged 16–26 (25). Therefore, early vaccination is crucial for maximizing protection in younger individuals, while the catch-up vaccination for 15–26 year-olds should be targeted with strategies that address the barriers preventing timely uptake, such as financial constraints and healthcare access.

Additionally, urban–rural and ethnic differences play a significant role in vaccine distribution (26–28). The study potentially substantiates that urban populations, advantaged by superior healthcare infrastructure and enhanced access to information, demonstrated elevated vaccination rates—an observation corroborated by research conducted in highly developed economies such as the United States, characterized by its substantial GDP (29, 30). In contrast, rural populations and ethnic minorities face considerable challenges, including limited access to healthcare resources, economic constraints, and lower levels of health literacy. These barriers have contributed to lower vaccination rates and a preference for the domestic bivalent vaccine (31). Moreover, cultural factors, such as language barriers and a reliance on traditional medicine, might further exacerbate these disparities (32). Ethnic minority communities, particularly those in remote areas, often face additional challenges, including skepticism toward medical interventions and a lack of familiarity with vaccination programs, all of which impede vaccine uptake.

The economic stratification of vaccine type preferences unveils China’s evolving health consumption paradigm. While middle-income groups exhibit rational cost–benefit calculations favoring domestic bivalent vaccines, the unexpected 28.5% 9-valent preference among lowest-income cohorts challenges conventional behavioral models. This anomaly reflects the emergence of “health investment families”—particularly student populations supported by intergenerational resource pooling (33, 34)—where aspirational health behaviors transcend current socioeconomic status. Simultaneously, social media’s dual role as information conduit and status-signaling platform accelerates demand differentiation, creating vaccine market segmentation that mirrors broader consumer trends in digital China (35, 36).

Three-dose regimen attrition (24.05% non-completion) exposes critical weaknesses in immunization service design. The temporal decay in adherence correlates not merely with logistical barriers but reflects fundamental cognitive biases in risk perception—a phenomenon where immediate costs outweigh abstract future benefits (37). This behavioral economics perspective necessitates reengineering vaccination systems through commitment devices and loss-framed incentives rather than conventional awareness campaigns.

To address these challenges, a multi-dimensional intervention system is necessary. First, a tiered subsidy scheme for low-income families should be designed (38, 39), linking the out-of-pocket cost for the 9-valent vaccine to income levels, while simultaneously implementing a bundled payment system for the three-dose regimen to improve completion rates. Second, an AI-based vaccination management system should be developed, utilizing social media to send personalized reminders, particularly establishing digital health records for the 9–26 age group (40). Furthermore, HPV vaccination should be integrated into the performance assessment system for primary healthcare, with rural doctors trained as community-level vaccine advocates (41). It is important to note that while this study’s retrospective design reveals macro-epidemiological patterns, it fails to capture the influence of micro-level mechanisms such as cultural taboos and doctor-patient interactions. Future research should use mixed-methods approaches to deconstruct the role of intersecting social identities, such as ethnicity and occupation, in shaping vaccination behaviors.

## 5 Conclusion

China’s HPV vaccination landscape in Tianjin reflects broader socio-economic inequalities across the country. By addressing age-related disparities, bridging the urban–rural divide, and implementing occupation-specific strategies, policymakers can transform cervical cancer prevention from a privilege into a universal safeguard. These efforts would not only promote health equity but also position China as a global leader in overcoming vaccine hesitancy—a critical challenge in an era marked by evolving pathogens and fragmented trust.

## Data Availability

The original contributions presented in the study are included in the article/supplementary material, further inquiries can be directed to the corresponding author.

## References

[1] Singh D Vignat J Lorenzoni V Eslahi M Ginsburg O Lauby-Secretan B. Global estimates of incidence and mortality of cervical cancer in 2020: a baseline analysis of the WHO global cervical cancer elimination initiative. Lancet Global Health. (2023) 11:e197–206. doi: 10.1016/S2214-109X(22)00501-0, PMID: 36528031 PMC9848409

[2] Sokale IO Oluyomi AO Montealegre JR Thrift AP. Racial/ethnic disparities in cervical cancer stage at diagnosis: mediating effects of neighborhood-level socioeconomic deprivation. Epidemiol Biomarkers Prev. (2023) 32:818–24. doi: 10.1158/1055-9965.EPI-23-0038 PMID: 37067295 PMC10233349

[3] Donkers H Bekkers R Massuger L Galaal K. The impact of socioeconomic deprivation on survival in cervical cancer patients: a systematic review. Lancet Global Health. (2019) 29:A210–A211. doi: 10.1136/ijgc-2019-ESGO.346

[4] Cham S Gamble C Rauh-Hain A Tergas A Hershman D Wright J. Disparities in cervical cancer incidence and neighborhood socioeconomic inequality in New York City. Gynecol Oncol. (2021) 162:S64. doi: 10.1016/S0090-8258(21)00763-0 PMC8777563 34817550

[5] Sung H Ferlay J Siegel RL Laversanne M Soerjomataram I Jemal A. Global cancer statistics 2020: GLOBOCAN estimates of incidence and mortality worldwide for 36 cancers in 185 countries. CA Cancer J Clin. (2021) 71:209–49. doi: 10.3322/caac.21660, PMID: 33538338

[6] Li P Liu Q Li W Liu Z Xing B Wu S. Characteristics of human papillomavirus infection among women with cervical cytological abnormalities in the Zhoupu District, Shanghai City, China, 2014–2019. Virol J. (2021) 18:51. doi: 10.1186/s12985-021-01518-y, PMID: 33685499 PMC7938559

[7] Wang WP An JS Yao HW Li N Zhang YY Ge L. Prevalence and attribution of high-risk HPV in different histological types of cervical cancer. Zhonghua Fu Chan Ke Za Zhi. (2019) 54:293–300. doi: 10.3760/cma.j.issn.0529-567x.2019.05.002, PMID: 31154709

[8] Paz-Zulueta M Álvarez-Paredes L Rodríguez-Díaz JC Parás-Bravo P Andrada Becerra ME Rodríguez Ingelmo JM. Prevalence of high-risk HPV genotypes, categorised by their quadrivalent and nine-valent HPV vaccination coverage, and the genotype association with high-grade lesions. BMC Cancer. (2018) 18:112. doi: 10.1186/s12885-018-4033-2, PMID: 29382323 PMC5791190

[9] Hariri S Bennett NM Niccolai LM Schafer S Park IU Bloch KC. Reduction in HPV 16/18-associated high grade cervical lesions following HPV vaccine introduction in the United States-2008-2012. Vaccine. (2015) 33:1608–13. doi: 10.1016/j.vaccine.2015.01.084, PMID: 25681664 PMC7522784

[10] Yakely A Oliveira C Niccolai L. Declining trends in anogenital warts since HPV vaccine introduction in a large urban health system, 2013-2017. Sex Transm Infect. (2019) 95:A79. doi: 10.1136/sextrans-2019-sti.111

[11] Fangjian G Cofie LE Berenson AB. Cervical cancer incidence in young U.S. females after human papillomavirus vaccine introduction. Am J Prev Med. (2018) 55:197–204. doi: 10.1016/j.amepre.2018.03.013, PMID: 29859731 PMC6054889

[12] Falcaro M Castañón A Ndlela B Soldan K Checchi M Lopez-Bernal J. The effects of the national HPV vaccination programme in England, UK, on cervical cancer and grade 3 cervical intraepithelial neoplasia incidence: a register-based observational study. Lancet. (2021) 398:2084–92. doi: 10.1016/S0140-6736(21)02178-4, PMID: 34741816

[13] La Torre G Balducci S Masala G. Estimating the impact of an organised screening programme on cervical cancer incidence: a 26-year study from northern Italy. Int J Cancer. (2019) 144:1017–26. doi: 10.1002/ijc.31806, PMID: 30120770

[14] You T Zhao X Pan C Gao M Hu S Liu Y. Informing HPV vaccine pricing for government-funded vaccination in mainland China: a modelling study. Lancet Reg Health West Pac. (2024) 52:101209. doi: 10.1016/j.lanwpc.2024.101209 39430124 PMC11489076

[15] National Bureau of Statistics. China statistical yearbook 2023. Beijing: China Statistics Press (2023).

[16] Brandt HM Footman A Adsul P Ramanadhan S Kepka D. Implementing interventions to start HPV vaccination at age 9: using the evidence we have. Hum Vaccin Immunother. (2023) 19:2180250. doi: 10.1080/21645515.2023.2180250, PMID: 36803261 PMC10026886

[17] Saxena K Kathe N Sardana P Yao L Chen YT Brewer NT. HPV vaccine initiation at 9 or 10 years of age and better series completion by age 13 among privately and publicly insured children in the US. Hum Vaccin Immunother. (2023) 19:2161253. doi: 10.1080/21645515.2022.2161253, PMID: 36631995 PMC9980633

[18] Xie P Zhao J Li X Zou X Liu G Han X. Preference for human papillomavirus vaccine type and vaccination strategy among parents of school-age girls in Guangdong province, China. Prev Med Rep. (2023) 36:102463. doi: 10.1016/j.pmedr.2023.102463, PMID: 37854667 PMC10580040

[19] Wang Z Wang J Fang Y Gross DL Wong MCS Wong ELY. Parental acceptability of HPV vaccination for boys and girls aged 9–13 years in China—a population-based study. Vaccine. (2018) 36:2657–65. doi: 10.1016/j.vaccine.2018.03.057, PMID: 29606519

[20] Essa-Hadad J Gorelik Y Vervoort J Jansen D Edelstein M. Understanding the health system barriers and enablers to childhood MMR and HPV vaccination among disadvantaged, minority or underserved populations in middle- and high-income countries: a systematic review. Eur J Pub Health. (2024) 34:368–74. doi: 10.1093/eurpub/ckad232, PMID: 38183166 PMC10990506

[21] Gautam N Dessie G Rahman MM Khanam R. Socioeconomic status and health behavior in children and adolescents: a systematic literature review. Front Public Health. (2023) 11:1228632. doi: 10.3389/fpubh.2023.1228632, PMID: 37915814 PMC10616829

[22] Cui M Wang Y Liu Z Liu C Niu T Zhou D. The awareness and acceptance of HPV vaccines among parents of primary and junior high school students in China: a meta-analysis. Infect Med (Beijing).. (2023) 2:273–82. doi: 10.1016/j.imj.2023.11.003 38205181 PMC10774669

[23] Gao M Hu S Zhao X You T Jit M Liu Y. Health and economic impact of delaying large-scale HPV vaccination and screening implementation on cervical cancer in China: a modelling study. Lancet Reg Health West Pac, (2023) 36:100768. doi: 10.1016/j.lanwpc.2023.100768 37547038 PMC10398607

[24] Hao S Wang C Liu S He J Jiang Y. HPV genotypic spectrum in Jilin province, China, where non-vaccine-covered HPV53 and 51 are prevalent, exhibits a bimodal age-specific pattern. PLoS One. (2020) 15:e0230640. doi: 10.1371/journal.pone.0230640, PMID: 32208459 PMC7313545

[25] Centers for Disease Control and Prevention (2024). Chapter 11: human papillomavirus. Available online at: https://www.cdc.gov/pinkbook/hcp/table-of-contents/chapter-11-human-papillomavirus.html

[26] Siu JY Fung TKF Leung LH. Social and cultural construction processes involved in HPV vaccine hesitancy among Chinese women: a qualitative study. Int J Equity Health. (2019) 18:147. doi: 10.1186/s12939-019-1052-9, PMID: 31533722 PMC6751778

[27] Yuan K Zhang P Yang M Zhang S. Vaccination prevalence and influencing factors of HPV vaccine among women in Tengzhou city, 2018–2020: a paired case-control study. Chin J Public Health.. (2021) 37:1746–50. doi: 10.11847/zgggws1134989

[28] Tsai Y Lindley MC Zhou F Stokley S. Urban-rural disparities in vaccination service use among low-income adolescents. J Adolesc Health. (2021) 69:114–20. doi: 10.1016/j.jadohealth.2020.10.021, PMID: 33288460 PMC8175462

[29] Lee M Gerend MA Boakye EA. Rural-urban differences in human papillomavirus vaccination among young adults in 8 US states. Am J Prev Med. (2021) 60:298–9. doi: 10.1016/j.amepre.2020.07.023, PMID: 33067069

[30] Polter EJ Christianson B Steinberg A Doan M Ljungman H Sundaram ME. Urban and rural healthcare providers’ perspectives on HPV vaccination in Minnesota. Hum Vaccin Immunother. (2023) 19:2291859. doi: 10.1080/21645515.2023.2291859, PMID: 38095606 PMC10730133

[31] Peterson CE Silva A Holt HK Balanean A Goben AH Dykens JA. Barriers and facilitators to HPV vaccine uptake among US rural populations: a scoping review. Cancer Causes Control. (2020) 31:801–14. doi: 10.1007/s10552-020-01323-y, PMID: 32537702

[32] Wang D Wu J Du J Ong H Tang B Dozier M. Acceptability of and barriers to human papillomavirus vaccination in China: a systematic review of the Chinese and English scientific literature. Eur J Cancer Care. (2022) 31:e13566. doi: 10.1111/ecc.13566, PMID: 35229931 PMC9287030

[33] Zastawna B Milewska A Załuska R Kozłowski R Zastawna M Marczak M. Analysis of parents’ attitudes and knowledge toward immunization and how these factors influence their decisions to vaccinate their children against human papillomavirus (HPV). Medicina. (2023) 59:1755. doi: 10.3390/medicina59101755, PMID: 37893473 PMC10608555

[34] Buller DB Pagoto S Henry K Berteletti J Walkosz BJ Bibeau J. Human papillomavirus vaccination and social media: results in a trial with mothers of daughters aged 14–17. Front Digital Health. (2021) 3:683034. doi: 10.3389/fdgth.2021.683034, PMID: 34713152 PMC8521953

[35] Tobaiqy M MacLure K. A systematic review of human papillomavirus vaccination challenges and strategies to enhance uptake. Vaccine. (2024) 12:746. doi: 10.3390/vaccines12070746, PMID: 39066384 PMC11281456

[36] Koskan A Cantley A Li R Silvestro K Helitzer D. College students’ digital media preferences for future HPV vaccine campaigns. J Cancer Educ. (2022) 37:1743–51. doi: 10.1007/s13187-021-02022-1, PMID: 33934288 PMC8088485

[37] Gaube S Lermer E Fischer P. The concept of risk perception in health-related behavior theory and behavior change. In: Raue M, Streicher B, Lermer E, eds. Perceived Safety: A Multidisciplinary Perspective. Risk Engineering series. 1st ed. Springer Cham. (2019) 101–18.

[38] Sloan K Shin M Palinkas LA Hudson SV Crabtree BF Cantor JC. Exploring HPV vaccination policy and payer strategies for opportunities to improve uptake in safety-net settings. Front Public Health. (2023) 11:1099552. doi: 10.3389/fpubh.2023.1099552, PMID: 37213634 PMC10192548

[39] Spencer JC Brewer NT Coyne-Beasley T Trogdon JG Weinberger M Wheeler SB. Reducing poverty-related disparities in cervical cancer: the role of HPV vaccination. Cancer Epidemiol Biomarkers Prev. (2021) 30:1895–903. doi: 10.1158/1055-9965.EPI-21-0307, PMID: 34503948 PMC8492489

[40] Dudeja N Khan T Varughese DT Abraham SG Ninan MM Prasad CL. Technologies for strengthening immunization coverage in India: a systematic review. Lancet Reg Health Southeast Asia. (2024) 23:100251. doi: 10.1016/j.lansea.2023.100251, PMID: 38404512 PMC10884965

[41] Murciano-Gamborino C Diez-Domingo J Fons-Martinez J PROTECT-EUROPE Consortium. Healthcare professionals’ perspectives on HPV recommendations: themes of interest to different population groups and strategies for approaching them. Vaccine. (2024) 12:748. doi: 10.3390/vaccines12070748, PMID: 39066386 PMC11281591

